# How evolutionary science can help us understand vaccine refusal in the COVID-19 pandemic

**DOI:** 10.1192/bjb.2022.36

**Published:** 2023-08

**Authors:** Annie Swanepoel, Riadh Abed, Muzaffer Kaser, Paul St John Smith

**Affiliations:** 1 North East London NHS Foundation Trust, Chelmsford, UK; 2 Retired, UK; 3 Cambridgeshire and Peterborough NHS Foundation Trust, Cambridge, UK; and University of Cambridge, UK

**Keywords:** Evolution, COVID-19, vaccine refusal, systemic racism, social deprivation

## Abstract

Unvaccinated people have a mortality rate from COVID-19 that is 32-fold that of fully vaccinated people. Yet, in the UK, more than 4% of adults have not accepted a vaccine to protect them against COVID-19 and at the time of writing only 73% of people were fully vaccinated. Psychological and societal factors underlying vaccine hesitation or refusal are complex. In this paper, we use evolutionary science to help explain how vaccine refusal can be the result of an historic adaptation to protect against the repetition of past trauma, including, for many, that of systemic racism and/or deprivation, and misguided attempt to preserve fertility. We discuss some resulting cognitive biases and conclude with recommendations for practice.

The COVID-19 pandemic has, at the time of writing, led to over 5 million deaths worldwide.^1^ This number would undoubtably have been significantly greater without the development of effective vaccines. The Office for National Statistics reported that the mortality rate among unvaccinated people is about 850/100 000, compared with 26/100 000 in fully vaccinated people.^2^ Yet more than 4% of adults in the UK are actively choosing not to be vaccinated.^3^ This is surprising, as the vaccines are effective, free, easily available and widely recommended. Over 138 million vaccine doses have been given in the UK, yet only 73% of the UK population is fully vaccinated and over 157 000 COVID-related deaths have been reported.^4^ Worldwide over 10 billion doses of the vaccine have been given and 53.7% of the world is fully vaccinated.^5^ Vaccines save lives and the majority of the population accept this. What needs to be explained is that a significant minority of the population refuse vaccination despite its clear benefits in terms of health and survival.

The UK data about unvaccinated people is clear: there is a preponderance of Black or Black British people, of whom 21% are unvaccinated, as are 14% of the Muslim community and 4% of White adults. Also, in communities with high levels of deprivation, the rate of nonimmunised people is 8%, whereas among the least deprived it is 2%.^3^ However, in studies, psychological measures of collectivism (the consideration of individual versus group benefits) and reactance (intolerance of others telling one what to do) outperform ethnic and financial status in predicting vaccine refusal.^6^ Research suggests that a distrust of authority is the best predictor of not being vaccinated.^7,8^

Psychiatrists are well placed to help understand the problem of lack of trust. We know that distrust in authority figures is usually based on past experiences. Historically, there have been many ethical violations in the healthcare of Black people, of which the Tuskegee experiment, in which people with syphilis were left untreated despite the availability of penicillin, is but one example.^9^ Also, we can speculate that the success of previous vaccinations against measles and polio has led to a loss of cultural memory of childhood deaths and disability and that vaccine refusal now feels less personally threatening.

Currently, deepening social inequality and deprivation understandably lead to decreasing trust in a government that is supposed to help the underprivileged, but does not do so.^9^

Where trust is lacking, conspiracy theories are more likely to arise and spread. A conspiracy is defined as a secret plan made by malevolent, powerful people. As there is plenty of evidence of this having happened in the past and a lived experience of being at the mercy of people in authority now, it is not surprising that conspiracy theories flourish. Social media allow like-minded people to find each other, which reaffirms group thinking. Not trusting people who have a track record of not having your best interests at heart is not pathological, but adaptive. Evolutionary science can help explain this.

## What we can learn from evolutionary science

The mark of evolutionary success is survival and reproduction. Natural selection describes the process by which those organisms that are most suited to their environment have the best chance to survive and reproduce and their genes are therefore propagated. Evolution has no foresight and only has the past to go on and thus does not prepare us for just one optimal future condition. On the contrary, there are often a wide range of possible environments and there are also many different strategies that can be successful. For example, in a benign environment, it makes sense to delay reproduction, have fewer children and invest more in each. This is typical of a ‘slow life history’. On the other hand, if the environment is harsh and survival is not certain, those people who follow a ‘fast life history’ and reproduce early and have more children have a greater chance of leaving living descendants.^10^

People who have been oppressed and whose fertility has been curtailed will be much more sensitive to threats to their future reproduction. This is the case for ethnic minorities and for people who live under conditions of high deprivation. Nesse described how the functioning of the anxiety system is akin to a smoke detector.^11^ It is evolutionarily sensible to overreact to avoid a potentially catastrophic danger (a false positive), rather than be complacent and die (a false negative) or be unable to leave descendants. Therefore, the widespread fear that the vaccine may reduce fertility^12^ is highly salient, despite the lack of scientific evidence to support this fear. It may, therefore, not be a coincidence that the vaccination rate in older people is close to 100%, whereas almost 1 in 10 of 22- to 25-year-olds are not vaccinated.^3^ Of course, a further explanation is that COVID-19 has a disproportionate impact on the elderly compared with the young.

Detecting and avoiding threats is a crucial skill shaped by natural selection. When this ability is triggered unnecessarily (false positive), it may be expressed as conspiratorial thinking and even paranoia. However, where there has been a proven threat in the past, humans have generally adapted to be more careful in future. Post-traumatic stress disorder (PTSD), with the symptoms of hypervigilance, avoidance of similar situations and re-experiencing of trauma, is, in moderate levels, evolutionarily adaptive in that it keeps us on our toes, keeps us safe and does not let us forget. This can help us avoid a future catastrophic danger and therefore allows us to survive and reproduce. However, PTSD can be excessive, as seen in clinical cases, being seen as an extreme of such an adaptation and a mismatch with modern environments.^13^

The role of widespread misinformation should also be considered. Ironically, even before the pandemic, social psychology researchers proposed that an ‘inoculation’ of facts in the era of fake news may be needed.^14^ Such an approach would build on trust and counteract unhelpful impulsive decisions in the context of emotionally salient information. Given the fast pace of changing information during the pandemic, people who have persistent trust issues may be drawn into more extreme views. A line of evidence showed that a shift to extreme views is associated with impulsive decisions based on imperfectly processed evidence.^15^ One might argue that vaccine misinformation hijacked the trust systems that pushed the limits of an adaptive system interfering with decision-making even in the face of compelling evidence of vaccine effectiveness. Widely available access to social media platforms, and the algorithms that potentiate continuous flow of misinformation, may also have played a role.

It is understandable that those who have suffered discrimination, oppression or racism from authorities in the past will be much more likely to suspect it will happen again. Interestingly, even some people who have not suffered discrimination have struggled with taking advice to get vaccine ‘boosted’ from politicians who have been mendacious over other matters. We need to understand how much more difficult it is for those people to trust healthcare professionals, especially those who have experienced recent or past intergenerational trauma from authority figures.

Recent research confirms that people are much more likely to be vaccinated if other people like them are vaccinated too. Evolutionary science also describes how it has been critically important in our evolutionary past to not be ostracised from our in-group, as that would have meant certain death. We are therefore adapted to fit in with our family and friends and find it very difficult to go against what they advise.^16^ This is especially relevant (and problematic) if our friends are against accepting the vaccine.

Lastly, evolutionary theory informs us about ‘trade-offs’: as life is rarely easy, we need to weigh up the risks and benefits to make decisions. These are mostly unconscious. We are the descendants of a long unbroken line of ancestors who survived and reproduced, and we have evolved to prioritise our own survival and that of our offspring.^10^ Therefore, we are vulnerable to cognitive biases if the information we are provided with does not match what we feel we need to achieve these aims (Box 1).

Box 1 Cognitive biases: examples that can arise in terms of vaccine refusal^17^

**Cognitive dissonance**

If the facts are in conflict with our opinions, we are more likely to discount the facts:

‘The healthcare professionals say the vaccine is safe, but my parents and friends say it's dangerous. I also think the pharmaceutical companies lie and are profiteering.’

**Omission bias**

Believing that a bad outcome is morally more reprehensible when caused by an action rather than an inaction:

‘If I allow my child to be immunised and she has side-effects, it is my fault. If she gets COVID and is ill, it is not my fault.’

**Confirmation bias**

Focusing on information that supports our pre-existing beliefs and ignoring any evidence presented to the contrary:

‘My aunt had COVID and was fine, so I'll be fine too – the newspapers exaggerate.’

**Availability heuristic**

Rare occurrences are newsworthy and memorable and seem to be much more prevalent than they are:

‘I've seen a lot about people getting clotting problems and heart problems from the vaccine. Therefore it is too dangerous for me or my children.’

**Illusory correlation**

Correlation is not causation – but may seem like it:

‘My cousin had the vaccine and then 6 months later had a miscarriage. I'm not risking it.’

## Where psychiatric skills can help

Psychiatrists are experts at diagnosing and managing mental disorder. In terms of vaccine refusal, it is very rare that overt psychopathology is the primary cause. One study demonstrated that some psychiatric patients have higher rates of vaccine refusal than the general population, especially those with PTSD, anxiety disorders and substance use disorders.^18^ However, another study demonstrated that vaccine hesitancy in psychiatric in-patients mapped more clearly on to educational and ethnic characteristics.^19^

The definition of a delusion requires that the belief is not one ordinarily accepted by other members of the person's culture or subculture and is particularly self-referential, which clearly does not apply to general vaccine refusal. Consequently, the main role for a psychiatrist should be in terms of applying legislation such as the Mental Capacity Act 2005 in England and Wales, designed for patients who have a disturbance of the mind or brain. Patients are thereby evaluated on their ability to understand, retain and weigh up the risks and benefits and communicate their decision. It is nevertheless important to remember that people are allowed to make unwise decisions.

The role of a psychiatrist must not lie in inappropriately forcing people to do something they do not want to do. The Mental Health Act 1983 clearly does not apply to people who simply refuse vaccines. What psychiatrists as doctors can do, though, is to help understand the patient's reluctance and to educate and persuade them to move in the direction that is in their best interests. Psychiatrists are also well placed to explore past experiences that could cause mistrust of the healthcare system, and other reasons for hesitancy, including needle phobia. As psychiatrists we need to be mindful, at least in theory, that some patients who have been forced to have injected medication (for example during rapid tranquillisation or depot antipsychotic medication) may have complex transference responses which may lead to our involvement being unintentionally counterproductive when we recommend vaccination.

## Problems that require a political solution

We have so far focused on the individual. However, it is clear that there are international forces at work that have a great influence on individuals that may surpass the effects of their in-group of family and friends. People who are ideologically motivated to be against vaccines are called anti-vaxxers. The anti-vaxxers are partly driven by people who are economically motivated by selling ineffective preparations that supposedly protect against COVID-19 or hasten recovery, or who earn substantial sums from social media sites through the traffic they attract to their sites.^17^ There are also anti-vaxxers who are politically motivated and who aim to destabilise countries and governments. A recent investigation showed that those spreading misinformation are ‘well financed, determined and disciplined’ and ‘actively working to sow doubts about the deadliness of COVID-19, vaccines and medical professionals’ integrity’.^20^ Manipulating people for political or economic gain is a part of international big business and is poorly regulated in online services. Scams and disinformation need to be combatted at a national level.

In the USA, political affiliation is also a major determinant of one's stance on vaccines, even stronger than ethnicity or socioeconomic status. A polarised political landscape may have contributed to extreme decisions that would not be possible in other circumstances.^21^

## Recommendations for practice

It is easier to blame an individual than to consider complex systemic effects – this is as true for obesity and child maltreatment as it is for people who refuse to accept the COVID-19 vaccine. Unfortunately, the natural protective effect that distrust in authority holds for people who have reason to feel that way can be co-opted and amplified by others who seek financial and/or political gain by exploiting that distrust.

As vaccine hesitancy is a complex, multifaceted problem, a one-size-fits-all solution will not be effective. Strategies that have been shown to increase vaccine uptake are:^22,23^

- listening to the individual and taking their concerns seriously and using motivational interviewing skills to help
- not engaging with misinformation online, as that can increase traffic and amplify it
- sharing good-quality information from organisations that are above distrusted authority figures, for example from the World Health Organization^24^ rather from than the national government
- encouraging scientists, faith leaders and healthcare professionals to share that they are vaccinated and that it is safe and to support others from their social and/or ethnic background to be able to do the same
- avoiding the imposition of mandatory vaccination programmes that may increase uptake in the short-term, but may cause a serious backlash against all vaccines in the longer term.^25^

## Conclusions

Psychiatrists should be well placed to understand and provide advice for tricky medical problems. Addressing vaccine refusal by considering (a) predisposing factors such as the intergenerational trauma of racism, (b) precipitating factors such as (untrue) accounts of vaccines affecting fertility and (c) avoiding perpetuating factors such as anti-vaxxer social media echo chambers can help us understand that vaccine refusal is a serious and complex problem needing a multipronged approach and prevention of knee-jerk simplistic reactions that are likely to backfire.

In the short term, we need to be respectful of people who are wary of being vaccinated and be careful not to add to their distrust of authority by being dismissive of them and confirming their mistrust in professionals. In the longer term, we need to advocate for better education to help people spot misinformation and for better social support to build trust in appropriate authority figures. Many polls have confirmed that doctors are highly trusted by the public – much more so than politicians.^26^ Let us use that trust in the best interests of the communities we serve.

## About the authors

**Annie Swanepoel** is a consultant child and adolescent psychiatrist working in Southend, Essex and Thurrock Children's and Adolescent's Mental Health Service (SET CAMHS), North East London NHS Foundation Trust (NELFT), Chelmsford, UK. **Riadh Abed** is a retired consultant general adult psychiatrist living in Sheffield, UK. **Muzaffer Kaser** is a consultant general adult and liaison psychiatrist working in Cambridgeshire and Peterborough NHS Foundation Trust and in the Department of Psychiatry at the University of Cambridge, UK. **Paul St John Smith** is a retired consultant general adult psychiatrist living in St Albans, UK.

## Data Availability

Data availability is not applicable to this article as no new data were created or analysed in this study.
